# Application of Auxiliary VerifyNow Point-of-Care Assays to Assess the Pharmacodynamics of RUC-4, a Novel αIIbβ3 Receptor Antagonist

**DOI:** 10.1055/s-0041-1732343

**Published:** 2021-09-28

**Authors:** Ohad S. Bentur, Jihong Li, Caroline S. Jiang, Linda H. Martin, Dean J. Kereiakes, Barry S. Coller

**Affiliations:** 1 Allen and Frances Adler Laboratory of Blood and Vascular Biology, Rockefeller University, New York, New York, United States; 2 The Rockefeller University Hospital, New York, New York, United States; 3 The Carl and Edyth Lindner Center for Research and Education at the Christ Hospital, Cincinnati, Ohio, United States

**Keywords:** STEMI, platelet aggregation inhibitors, point-of-care testing, platelet glycoprotein GPIIb-IIIa complex, acute myocardial infarction

## Abstract

**Introduction** Prehospital therapy of ST-elevation myocardial infarction (STEMI) with αIIbβ3 antagonists improves clinical outcomes, but they are difficult to use in prehospital settings. RUC-4 is a novel αIIbβ3 antagonist being developed for prehospital therapy of STEMI that rapidly achieves high-grade platelet inhibition after subcutaneous administration. Standard light transmission aggregometry (LTA) is difficult to perform during STEMI, so we applied VerifyNow (VN) assays to assess the pharmacodynamics of RUC-4 relative to aspirin and ticagrelor.

**Methods** Blood from healthy volunteers was anticoagulated with phenylalanyl-prolyl-arginyl chloromethyl ketone (PPACK) or sodium citrate, treated in vitro with RUC-4, aspirin, and/or ticagrelor, and tested with the VN ADP + PGE _1_ , iso-TRAP, and base channel (high concentration iso-TRAP + PAR-4 agonist) assays. The results were correlated with both ADP (20 µM)-induced LTA and flow cytometry measurement of receptor occupancy and data from individuals treated in vivo with RUC-4.

**Results** RUC-4 inhibited all three VN assays, aspirin did not affect the assays, and ticagrelor markedly inhibited the ADP + PGE _1_ assay, slightly inhibited the iso-TRAP assay, and did not inhibit the base channel assay. RUC-4's antiplatelet effects were potentiated in citrate compared with PPACK. Cut-off values were determined to correlate the results of the VN iso-TRAP and base channel assays with 80% inhibition of LTA.

**Conclusion** The VN assays can differentiate the early potent anti-αIIbβ3 effects of RUC-4 from delayed effects of P2Y12 antagonists in the presence of aspirin. These pharmacodynamic assays can help guide the clinical development of RUC-4 and potentially be used to monitor RUC-4's effects in clinical practice.

## Introduction

Rapid reperfusion of the infarct-related artery is the best method to reduce the morbidity and mortality of ST segment elevation myocardial infarction (STEMI). 1 2 The earlier therapy is initiated the greater the likelihood that the thrombi will be platelet-rich and dynamic, 3 offering the best opportunity for pharmacological reperfusion.

Studies of oral P2Y12 antagonists administered along with aspirin at the first point of medical care have been disappointing, 4 5 6 in part because these agents require several hours to reach maximal effect in STEMI, especially when co-administered with opioids, even when crushed to speed absorption. 7 8 9 10 In contrast, two meta-analyses of studies of early intravenous therapy with the αIIbβ3 (GPIIb/IIIa) antagonists demonstrated significantly increased perfusion of the infarct-related artery at the time of percutaneous coronary artery intervention (PCI), even with concurrent clopidogrel therapy, more rapid ST-segment elevation resolution, higher left ventricular ejection fraction, and lower 6-month and long-term mortality. 11 12 13 14 15 16 17 18 19 However, these agents require bolus intravenous administration followed by a continuous infusion. Thus, there is a need in the prehospital therapy of STEMI 20 for a rapidly acting αIIbβ3 antagonist that can be administered subcutaneously (SC) and rapidly achieve high-grade inhibition of platelet function. To decrease the hemorrhagic risk, it would be desirable for the drug's antiplatelet effects to diminish as the antiplatelet effects of the commonly co-administered oral P2Y12 antagonists reach their maximal effects.

RUC-4 is a novel second-generation small-molecule αIIbβ3 antagonist. It has a unique mechanism of action that locks the receptor in its inactive conformation. 21 22 23 24 In a Phase 1 study conducted in healthy volunteers and stable coronary artery disease patients on aspirin, RUC-4 produced high grade inhibition of ADP (20 μM)-induced platelet aggregation as measured by light transmission aggregometry (LTA) within 15 minutes of SC administration, with rapid return of platelet function over the next 1–2 hours. 25 LTA is considered the established gold standard assay to assess the potency of antiplatelet agents because of its association with clinical outcomes. 19 26 27 28 29 30 31

As part of RUC-4's further development in Phase 2 studies of patients with STEMI undergoing emergency PCI, where it is impractical to perform multiple LTA assays, it would be useful to have an easy to perform automated assay to monitor the pharmacodynamics (PD) of the drug's effect and correlate the results with the drug's pharmacokinetics. It is important, however, that the assay correlates with the LTA assay. Moreover, since prehospital treatment with a P2Y12 antagonist is the standard of care in locations where RUC-4 is being tested, 2 it would be best to have an assay that can differentiate the effects of RUC-4 from those of the P2Y12 antagonist. In addition, near “real-time” data on the extent of RUC-4-mediated platelet inhibition on arrival in the cardiac catheterization laboratory may be important for the interventionalist to optimize additional adjunctive therapy, or to decide on the feasibility of emergency surgery.

To address this need, we evaluated the potential use of the VerifyNow (VN) assays, which are whole-blood, cartridge-based, and automated, and produce results within 15 minutes of blood draw. 32 Commercially available VN assays are designed to study the antiplatelet effects of aspirin (VN Aspirin cartridge; arachidonic acid activator) or P2Y12 antagonists (VN PRUTest cartridge and VN P2Y12 cartridge; ADP + PGE _1_ activator/inhibitor combination). A previously available assay for αIIbβ3-mediated platelet inhibition (VN GPIIb/IIIa cartridge) was withdrawn from the market, but the modified thrombin receptor activating peptide (iso-TRAP) used in that cartridge is also included in a separate reaction chamber (in addition to the ADP + PGE _1_ reaction chamber) in both the PRUTest and P2Y12 cartridges. 33 34 35 36 37 In addition, a ‘base channel’ reaction chamber in which iso-TRAP at higher concentrations is combined with a PAR-4 activating peptide (PAR-4 AP) to achieve potent activation that can overcome the effects of P2Y12 antagonists, is available in the P2Y12 assay cartridge. 34 35 37 To differentiate RUC-4's effects from the effects of P2Y12 antagonists and aspirin, we analyzed the effects of RUC-4, ticagrelor, and aspirin, alone or in combination, on the three different channels.

The choice of anticoagulant is important in assessing the impact of RUC-4 and other αIIbβ3 antagonists. Citrate is the most commonly employed anticoagulant for measuring platelet function, 39 but because it chelates divalent metal ions to below physiologic levels, it enhances the antiplatelet effects of eptifibatide and tirofiban measured by LTA relative to the effects when using anticoagulants that do not chelate divalent cations, such as heparin and phenylalanyl-prolyl-arginyl chloromethyl ketone (PPACK). 26 39 The use of citrate in early studies of these drugs resulted in an overestimation of their antiplatelet effects and the selection of drug doses that were inadequate to achieve the desired in vivo antithrombotic effect, followed by disappointing clinical efficacy. Adjusting the doses based on studies employing PPACK as the anticoagulant and using 20 μM ADP as the agonist resulted in selection of higher doses that proved clinically effective. 26 We previously showed that citrate anticoagulation also enhances RUC-4's measured antiplatelet effects. 24 As a result, in the Phase 1 study of RUC-4, LTA was performed with blood anticoagulated with PPACK. 25

In the present study, we endeavored to study the effect of RUC-4 and the P2Y12 antagonist ticagrelor *in vitro* using the ADP + PGE _1_ , iso-TRAP, and base channel assays in the PRUtest and P2Y12 cartridges. We also studied the effects of different anticoagulants and aspirin on the results, and then compared the data with a measure of receptor occupancy based on competitive binding of RUC-4 and the activation-dependent monoclonal antibody (mAb) PAC1. 41 Finally, we compared these data to those from patients with stable coronary artery disease on aspirin enrolled in the Phase 1 study, who also had measurements of their ADP-induced platelet aggregation by LTA using PPACK-anticoagulated blood.

## Methods

### *In Vitro* Studies

#### Blood Collection

All *in vitro* studies were approved by the Rockefeller University Institutional Review Board (IRB) and all blood donors gave informed consent. Blood from healthy volunteers, who were not taking aspirin, or from volunteers who were taking aspirin that was prescribed for primary or secondary prevention, was obtained via venipuncture with a 19-gauge needle. Blood was anticoagulated with 0.1 volume 3.2% sodium citrate (final concentration 0.32%) or 0.1 volume 1 mM PPACK (final concentration 100 µM) contained in the syringe. Anticoagulated blood to be used for the VN assays (Instrumentation Laboratories, Bedford, MA) was then distributed into 2-mL vacuum tubes (Greiner Bio-One Vacuette, Monroe, NC) that were designed for use with the VN assays. These tubes are supplied with a citrate anticoagulant, and so they were washed with running water 3 times and dried before being used in this study. Whole blood platelet counts and hematocrits were obtained with an automated counter (ADVIA 120 Hematology System, Siemens, Malvern, PA).

#### Preparation of RUC-4 and Ticagrelor-treated Whole Blood for VN Assays

50 µL of RUC-4 or ticagrelor stock solution (see Supplementary Material ) was added to 2 mL of whole blood and VN testing was performed after incubation for 10 minutes at room temperature for RUC-4 and 30 minutes at 37°C for ticagrelor. 42 All samples were tested within 3 hours of blood drawing.

#### VN Assays

In all the VN assays, blood is drawn from the blood collection tube into a cartridge, and then dispensed into separate reaction chambers containing fibrinogen-coated beads and an activator. 32 The PRUTest cartridge has two active reaction chambers, allowing it to conduct both the ADP + PGE _1_ and iso-TRAP assays on the same blood sample. The ADP + PGE _1_ reaction chamber contains ADP at 20 µM and prostaglandin E _1_ (PGE _1_ ) at 0.02 µM final concentrations; PGE _1_ is added to ADP in the ADP + PGE _1_ reaction chamber to increase the specificity of the results for P2Y12-mediated aggregation. 33 The iso-TRAP channel contains a final concentration of iso-TRAP of 3–4 µM. 32 34 The P2Y12 cartridge contains in addition to the ADP + PGE _1_ and iso-TRAP channels, the base channel, which contains 20 µM iso-TRAP + 800 µM PAR-4 AP. 34 35

We also utilized the VN Aspirin cartridge, whose reaction chamber contains arachidonic acid at a final concentration of 1 mM, to assess whether blood donors' platelets demonstrated an aspirin effect. 33

When activated with any of these agonists, platelets bind to the fibrinogen-coated beads and agglutination of the beads and platelets occurs in proportion to the number of available activated platelet receptors. This results in an increase in light transmission, which is reported in arbitrary reaction units. The VN PRUTest and P2Y12 cartridge assays currently only report the results from the ADP + PGE _1_ reaction chamber, with the results of the iso-TRAP assay used for internal quality control. 35 36 The results of the base channel in the P2Y12 cartridge are not reported or used for quality control. Whereas P2Y12 antagonists inhibit the ADP + PGE _1_ assay but have little or no inhibitory effects on the iso-TRAP assay, 38 43 the instrument reports an error when there is significant inhibition of the iso-TRAP assay. 34 35 Thus, blood treated with αIIbβ3 antagonists, which are potent enough to inhibit the iso-TRAP assay, cannot be tested with the PRUTest or P2Y12 cartridges when the instrument is used with its default settings. However, by connecting the VN instrument to a computer, we were able to collect raw data from all of the reaction chambers of the cartridges and process them by calculating the maximum change in light transmission from the beginning to the end of the assay run time. We expressed the results as reaction units and calculated the percentage inhibition of the assay relative to the control using the equation [1 -)test sample maximum change in light transmittance/control sample maximal change in light transmittance)] X 100.

#### Correlation of Percentage of αIIbβ3 Receptors Occupied by RUC-4 Determined by mAb PAC1 Binding with Both VN Assays and LTA

PAC1 is an IgM mouse mAb that selectively binds to activated αIIbβ3 in the same region occupied by RUC-4. 41 As a result, PAC1 will only bind to activated receptors that are not already occupied by RUC-4, providing information on the percentage of receptors occupied by RUC-4. PPACK-anticoagulated blood was treated with aspirin (0.3 mM) and RUC-4 (concentrations between 0.01 and 0.63 µM). After incubation, aliquots of the samples were tested in the VN assays used for preparing platelet-rich plasma (PRP) for LTA and flow cytometry (see Supplementary Material ).

#### LTA Studies

Platelet aggregation was initiated by adding 25 μL of agonist to 225 µL of PRP and monitored in a PAP-8E aggregometer (BioData Corporation, Horsham, PA) at 37°C with stirring. An agonist working stock solution of 200 µM ADP was prepared with sterile 0.9% saline. Platelet aggregation was quantified based on the instrument's measurement of the primary slope (PrSl) of aggregation, which is a measure of the change in light transmission per unit time sustained over at least a 15 second period. The percentage inhibition of the PrSl relative to the control value was calculated as [1-(test PrSl/control PrSl)] X 100. This measure was selected to avoid the need to choose an arbitrary time point for the comparison to baseline, or using the maximal aggregation, which can occur at different time points. Nonetheless, the PrSl correlates well with results based on maximal aggregation and final aggregation in the *in vitro* studies ( Supplementary Figure S1 ) and maximal aggregations in the in vivo studies. 24

### In Vivo Studies

The Phase 1 dose escalation safety and tolerability study of RUC-4 (NCT03844191) was approved by the IRB at The Christ Hospital, Cincinnati, OH. Healthy volunteers or patients with stable coronary artery disease receiving aspirin were enrolled following obtaining informed consent. 25 RUC-4 or placebo was administered SC in the deltoid region using a 25-gauge, ⅝ inch needle attached to a 1 mL syringe. A subgroup of patients with stable coronary artery disease taking aspirin who received RUC-4 at a dose of 0.075 mg/kg, had blood collected and prepared for both LTA studies as described 25 and for VN testing. The latter was collected with a 21-gauge needle into 2-mL sodium citrate vacuum tubes (final concentration 0.32%; Greiner Bio-One Vacuette). Platelet counts and hematocrits in whole blood and PRP were obtained with an automated counter (Sysmex poc-H100i, Sysmex, Lincolnshire, IL). RUC-4 levels were assayed on 1 mL samples of whole blood as described. 25

### Data Analysis

See Supplementary Material .

## Results

### *In Vitro* Studies of RUC-4

Whole blood samples for the *in vitro* studies were obtained from 15 healthy volunteers (8 males, 7 females) of whom 6 were taking aspirin at a dose of 81 to 325 mg/day. The mean platelet count (±SD) was 214 (±47) × 10 ^3^ platelets/µL. The antiplatelet effect of aspirin in the 6 participants who were taking aspirin was confirmed with the VN Aspirin assay, with those taking aspirin having values of 401–435 reaction units, all of which are below the cut-off value of 550 reaction units and thus consistent with aspirin-induced inhibition of platelet function, and those not taking aspirin having values of 670-771.

### RUC-4 Inhibits Both VN PRUTest Assays (iso-TRAP, ADP + PGE _1_ ) in a Concentration-dependent Manner, with Citrate-anticoagulated Blood more Sensitive to Inhibition than PPACK-anticoagulated Blood, and the ADP + PGE _1_ Assay more Sensitive to Inhibition than the iso-TRAP Assay

**Fig. 1:**
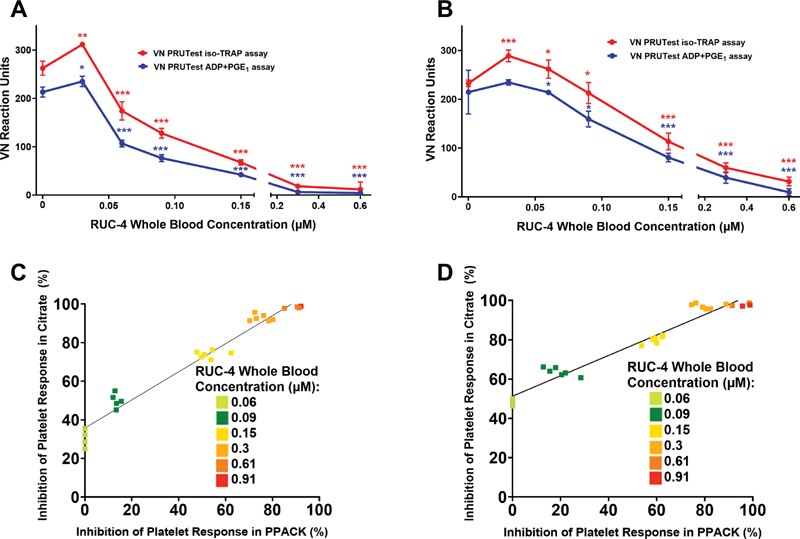
In vitro RUC-4 concentration-response in inhibiting the VN iso-TRAP and ADP + PGE _1_ assays with blood from healthy volunteers not taking aspirin and anticoagulated with sodium citrate or PPACK. ( **A** ) Concentration-response curve in citrate-anticoagulated blood ( *n* = 7), data are mean ± SD. ( **B** ) Concentration-response curve in PPACK-anticoagulated blood ( *n* = 7), data are mean ± SD. ( **C** ) Percentage inhibition of response of iso-TRAP assay in PPACK- versus citrate-anticoagulated blood ( *n* = 6; 25 measurements; R ^2^ = 0.96; *p* < 0.001). ( **D** ) Percentage inhibition of response of ADP + PGE _1_ assay with PPACK- versus citrate-anticoagulated blood ( *n* = 6; 25 measurements; R ^2^ = 0.95; *p* < 0.001). * *p* < 0.05 compared with control, ** *p* < 0.01 compared with control, *** *p* < 0.001 compared with control. VN = VerifyNow.

With citrate-anticoagulated blood, the VN PRUTest cartridge iso-TRAP assay reported an 18% increase in reaction units at the lowest concentration tested compared with baseline ( *p* < 0.01) and then progressive and marked inhibition compared with baseline ( *p* < 0.001) with higher concentrations ( Fig. 1A ). The ADP + PGE _1_ assay reported a 10% increase at the lowest concentration compared with baseline ( *p* < 0.02) with progressive dramatic inhibition compared with baseline with increasing RUC-4 concentrations ( *p* < 0.001). With PPACK-anticoagulated blood, the responses were qualitatively similar, with the increase in reaction units with iso-TRAP extending to one higher concentration, and with a progressive decrease in response at higher concentrations ( Fig. 1B ). The slope of the concentration-response inhibition with PPACK-anticoagulated blood was less steep than with citrate-anticoagulated blood for both assays at RUC-4 concentrations between 0.03 and 0.06 μM ( Fig. 1A,B ). For example, at 0.06 µM RUC-4 there was no inhibition of the iso-TRAP assay with PPACK-anticoagulated blood compared with ∼30% inhibition with citrate-anticoagulated blood. Similar results were observed with the ADP + PGE _1_ assay. Because of these anticoagulant-dependent differences, the IC _50_ s of RUC-4 were ∼40% lower with citrate-anticoagulated blood compared with PPACK-anticoagulated blood ( Table 1 ). There was, however, a close correlation between the results with the different anticoagulants with both assays at RUC-4 concentrations above 0.06 µM ( Fig. 1C,D ; iso-TRAP: R ^2^ = 0.96, *p* < 0.001; ADP + PGE _1_ : R ^2^ = 0.95, *p* < 0.001). Based on the greater sensitivity of the assays to RUC-4 at low concentrations in citrate-anticoagulated blood and the commercial availability of vacuum tubes for the VN assays containing citrate but not PPACK, we performed additional studies with citrate-anticoagulated blood.

**Table 1:** IC _50_ s (mean ± SD) of RUC-4 in PPACK- and sodium citrate-anticoagulated blood from volunteers who were either not taking or taking aspirin

VN PRUTest assay	IC _50_ (µM)			p value	
	PPACK No aspirin (1)	Citrate No aspirin (2)	Citrate Aspirin (3)	(1) vs (2)	(2) vs (3)
iso-TRAP	0.14 ± 0.01	0.09 ± 0.01	0.10 ± 0.02	<0.003	0.15
ADP + PGE _1_	0.12 ± 0.01	0.07 ± 0.01	0.08 ± 0.02	<0.03	0.19
p value	0.08	<0.004	<0.001		

Abbreviations: IC _50_ , Half maximal inhibitory concentration; VN, VerifyNow.

With citrate-anticoagulated blood, the percent inhibition by RUC-4 correlated closely between the ADP + PGE _1_ and iso-TRAP assays (R ^2^ = 0.97, *p* < 0.001), but the ADP + PGE _1_ assay was more sensitive as judged by the deviation from the line of identity at lower concentrations ( Supplementary Figure S2 ), with the IC _50_ ∼20% lower than that with the iso-TRAP assay ( Table 1 , *p* < 0.004).

### Aspirin does not Affect RUC-4's Inhibition of the VN PRUTest Assays

Since aspirin is the standard of care for STEMI and will be administered along with RUC-4, we tested the effect of aspirin by comparing the *in vitro* concentration-response to RUC-4 in blood obtained from donors who either were not taking aspirin or taking aspirin. The RUC-4 concentration-response curves ( Supplementary Figure S3 ) in citrate-anticoagulated blood from individuals who were taking aspirin had similar patterns to those from individuals who were not taking aspirin. Accordingly, the mean IC _50_ s of RUC-4 in citrate-anticoagulated blood were similar in both assays in individuals who were taking aspirin vs individuals who were not taking aspirin ( Table 1 : *p* = 0.15 in the iso-TRAP assay and *p* = 0.19 in the ADP + PGE _1_ assay).

### RUC-4 Produces Concentration-dependent Occupancy of Platelet αIIbβ3 Receptors that Correlates with Decreased Platelet Aggregation and VN PRUTest Assays' Reaction Units

**Fig. 2:**
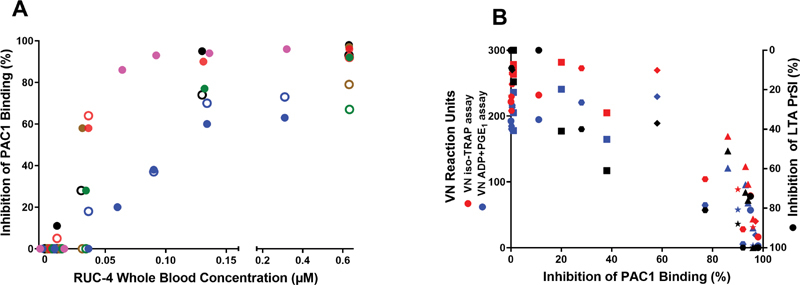
Correlation of PAC1 binding, reflecting the αIIbβ3 receptors not occupied by RUC-4, with RUC-4 whole blood concentration and inhibition of the VN and LTA assays in blood anticoagulated with PPACK and treated with aspirin. ( **A** ) Percentage inhibition of fluorescently-labeled PAC1 binding to ADP-activated platelets as a function of RUC-4 whole blood concentration. RUC-4 was added to whole blood and then both whole blood (open circles; *n* = 5; 24 measurements) and PRP prepared from that blood (filled circles; *n* = 6; 30 measurement) were activated with ADP (20 µM) and analyzed by flow cytometry. Each color signifies a different volunteer. ( **B** ) Percentage inhibition of PAC1 binding to ADP-activated platelets in PRP versus the 2 different VN assays (left axis) and inhibition of ADP (20 µM)-induced LTA PrSl (right axis). Each shape signifies a different volunteer, each color signifies a different assay. VN = VerifyNow; LTA = Light transmission aggregometry; PRP = Platelet-rich plasma; PrSl = Primary slope.

We previously reported that incubating whole blood with increasing concentrations of RUC-4 results in a decrease in the ability of PAC1 to bind to ADP-activated platelets, reflecting increased occupancy of αIIbβ3 receptors. 24 In the current study we assessed the effect of RUC-4 on PAC1 binding in parallel studies with both whole blood and PRP and correlated the results with the effects of RUC-4 on the VN PRUTest iso-TRAP and ADP + PGE _1_ assays and on ADP (20 µM)-induced LTA of PRP ( Fig. 2 ). As receptor occupancy increased, there was increased inhibition of the VN assay and aggregation measured by LTA ( Fig. 2B ). When PRP PAC1 binding was inhibited by 80% or more, the iso-TRAP assay was inhibited by 71% (71 ± 49 reaction units vs 242 ± 25 reaction units at baseline; *p* < 0.001), the ADP + PGE _1_ assay was inhibited by 77% (46 ± 4 reaction units vs 199 ± 11 reaction units at baseline; *p* < 0.001), and LTA was inhibited by 86 ± 17%.

### In Vivo Studies of RUC-4

#### In Vivo Administration of RUC-4 to Patients with Stable Coronary Artery Disease on Aspirin Results in Time-dependent Inhibition of Platelet Function Measured by ADP-induced LTA and Both the iso-TRAP and ADP + PGE _1_ VN Assays

Participants with stable coronary artery disease on aspirin in the Phase 1 study of RUC-4 ( *n* = 11; mean age 66 ± 7 years, 58% male, mean weight 87 ± 23 kg) who received 0.075 mg/kg of RUC-4 SC had blood drawn in PPACK (for LTA studies) or into vacuum tubes containing sodium citrate (for VN studies) at timed intervals.

**Fig. 3:**
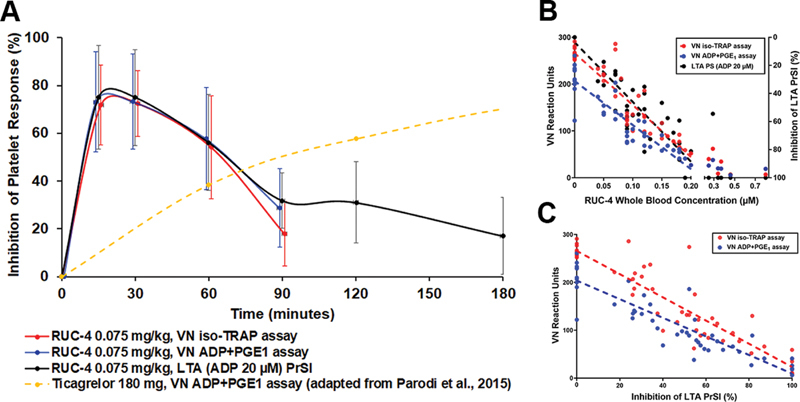
Inhibition of platelet function measured by the VN iso-TRAP and ADP + PGE _1_ assays of citrate-anticoagulated blood and ADP-induced LTA of PPACK-anticoagulated blood after RUC-4 subcutaneous administration to humans, and comparison with effect of ticagrelor after oral administration of crushed tablets to STEMI patients reported by Parodi et al., J Am Coll Cardiol 2015; 65: 511–512 (data extracted from figure using WebPlotDigitizer). ( **A** ) Inhibition of platelet responses as a function of time after administration of either RUC-4 (mean ± SD; *n* = 11, 50 measurements) or ticagrelor (VN ADP + PGE _1_ ; *n* = 41). ( **B** ) RUC-4 whole blood concentration versus VN reaction units (left axis) and inhibition of ADP (20 µM)-induced LTA PrSl (right axis) (linear regression lines: iso-TRAP assay: R ^2^ = 0.83; *p* < 0.001; ADP + PGE _1_ assay: R ^2^ = 0.81; *p* < 0.001; LTA: R ^2^ = 0.83; *p* < 0.001. ( **C** ) Inhibition of LTA PrSl versus VN reaction units (linear regression lines: iso-TRAP assay: R ^2^ = 0.84, *p* < 0.001; ADP + PGE _1_ assay: R ^2^ = 0.83; *p* < 0.001). VN = VerifyNow; LTA = Light transmission aggregometry; PrSl = Primary slope; PRP = Platelet- rich plasma.

The inhibition of ADP-induced LTA as a function of time after RUC-4 administration is shown in Fig. 3A (black line) and is notable for the onset of high-grade inhibition of aggregation by 15 minutes, followed by sustained high-grade inhibition at 30 minutes, and then a return toward baseline over the next two hours. The results of both the VN PRUTest iso-TRAP (red line) and ADP + PGE _1_ (blue line) assays at the first four time points are superimposed on the results of the ADP-induced LTA data and show a striking correlation during the first 3 time points. At the fourth time point, the iso-TRAP assay reported less inhibition than the ADP + PGE _1_ assay, reflecting the greater sensitivity of the latter to lower concentrations of RUC-4.

#### Inhibition of Both the iso-TRAP and ADP + PGE _1_ VN Assays Correlates with RUC-4 Blood Concentrations

Blood concentration of RUC-4 up to 0.2 µM correlated closely with inhibition of both VN assays ( Fig. 3B ; iso-TRAP assay: R ^2^ = 0.83, *p* < 0.001; ADP + PGE _1_ assay: R ^2^ = 0.81, *p* < 0.001). At concentrations above 0.2 µM the inhibition of the VN assays leveled off at 91 ± 8% for the iso-TRAP assay and 91 ± 4% for the ADP + PGE _1_ assay.

#### VN iso-TRAP Assay Reaction Units Correlate with LTA-measured Platelet Aggregation in Blood from Individuals Treated with RUC-4

Since RUC-4 will be administered prehospital in its clinical trials, the first VN assay will be obtained after it is administered. As a result, no baseline value will be available for comparison. Therefore, we assessed the relationship between inhibition of ADP-induced LTA and the absolute reaction units reported by the VN assays and found a close correlation at all time points with each assay ( Fig. 3C , iso-TRAP assay: R ^2^ = 0.84, *p* < 0.001, ADP + PGE _1_ assay: R ^2^ = 0.83, *p* < 0.001). When ADP-induced LTA was inhibited by ≥80%, the mean (±SD) VN reaction units measured with the iso-TRAP and ADP + PGE _1_ assays were 43 ± 38 and 31 ± 23, respectively.

#### Receiver Operating Characteristic Curve Analysis for Identifying the Optimal Cut-off Point for Monitoring RUC-4 Therapy with the VN Assays

**Fig. 4:**
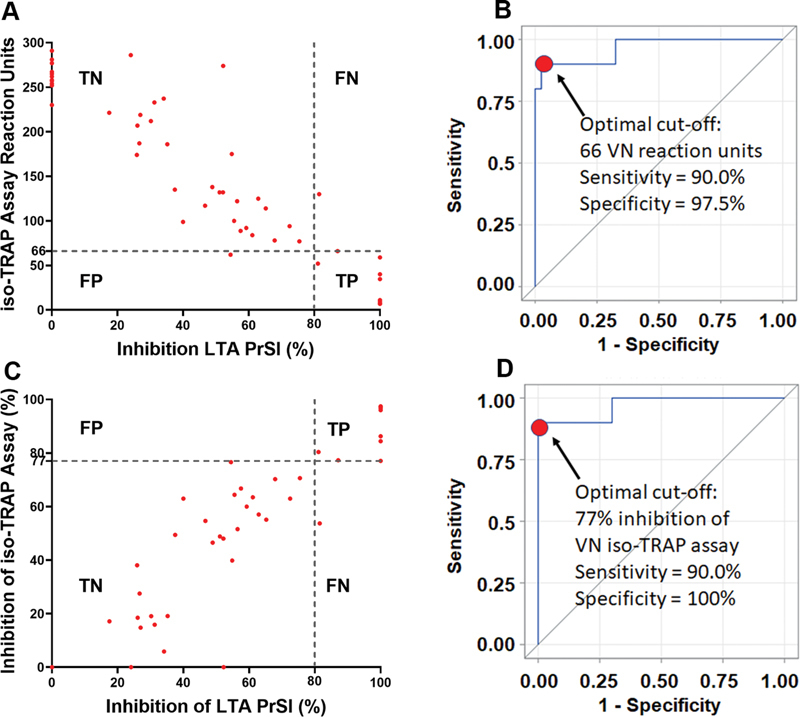
ROC curves identifying the VN PRUTest iso-TRAP assay reaction units that correspond to 80% or greater inhibition of the PrSl of ADP (20 µM)-induced LTA in patients on aspirin treated with RUC-4. ( **A** ) Reaction unit scatter plot. ( **B** ) Reaction unit ROC curve, identifying the optimal cut-off value as 66 reaction units. ( **C** ) Percentage inhibition scatter plot. ( **D** ) Percentage inhibition ROC curve, identifying the optimal cut-off as 77%. LTA was performed with PRP prepared from PPACK-anticoagulated blood, VN testing was performed with citrate-anticoagulated blood. *n* = 11 donors, 50 measurements. ROC = Receiver operating characteristic; VN = VerifyNow; PrSl = Primary Slope; LTA = Light transmission aggregometry; PRP = Platelet-rich plasma.

We used ROC curves of the absolute reaction units and the percentage inhibition of the VN assays to identify the optimal cut-off points for assessing the PD of RUC-4, with the binary classifier set at 80% inhibition of ADP-induced LTA. Correlated observations within subjects were accounted for by using a mixed effects logistic regression model. The Area Under the Curve (AUC) for the ROC curves was 0.97 (95% CI 0.9–1.0). The optimal iso-TRAP assay reaction unit cut-off point in citrate-anticoagulated blood for detecting ≥80% inhibition of LTA-measured platelet aggregation in PRP prepared from PPACK-anticoagulated blood was ≤66 reaction units (sensitivity = 90.0%, specificity = 97.5%), which corresponded to ≥77% inhibition (sensitivity = 90.0%, specificity = 100%) ( Fig. 4 ).

#### Hematocrit did not Significantly Affect either VN Assay

A low hematocrit has been associated with higher reaction unit values with both the ADP + PGE _1_ 44 45 46 47 48 49 50 and iso-TRAP 49 50 assays in blood samples from untreated individuals and individuals treated with clopidogrel. In our study, the mean (±SD) hematocrits at baseline in the *in vitro* and *in vivo* cohorts were 42 ± 4% (range: 34–51) and 42 ± 3% (range: 35–47), respectively. The plot of baseline readings of the VN assays vs hematocrit showed minor positive correlations (iso-TRAP assay: R ^2^ = 0.13, *p* = 0.08; ADP + PGE _1_ assay: R ^2^ = 0.16, *p* < 0.04) and thus we did not observe an inverse relationship between hematocrit and VN reaction units across the range of hematocrits in the individuals we studied ( Supplementary Figure S4 ).

### *In Vitro* Studies of Ticagrelor

#### RUC-4's Pharmacodynamics Complement Those of Ticagrelor

Fig. 3A shows the VN PRUTest ADP + PGE _1_ assay results in the Phase 1 study as a function of time after RUC-4 administration superimposed on the results reported by Parodi et al. using the VN ADP + PGE _1_ assay in their study of 41 patients with STEMI who received a 180 mg loading dose of ticagrelor as crushed tablets. 7 Thus, if given together, our data suggest that RUC-4's effects will begin to wear off as the effects of ticagrelor become more apparent, with the latter beginning to plateau after approximately two hours.

#### Ticagrelor Produces Less than 20% Inhibition of the VN iso-TRAP Assay at Concentrations that Inhibit the ADP + PGE _1_ Assay by 80% or More; the VN Base Channel Assay is Unaffected by Ticagrelor

**Fig. 5:**
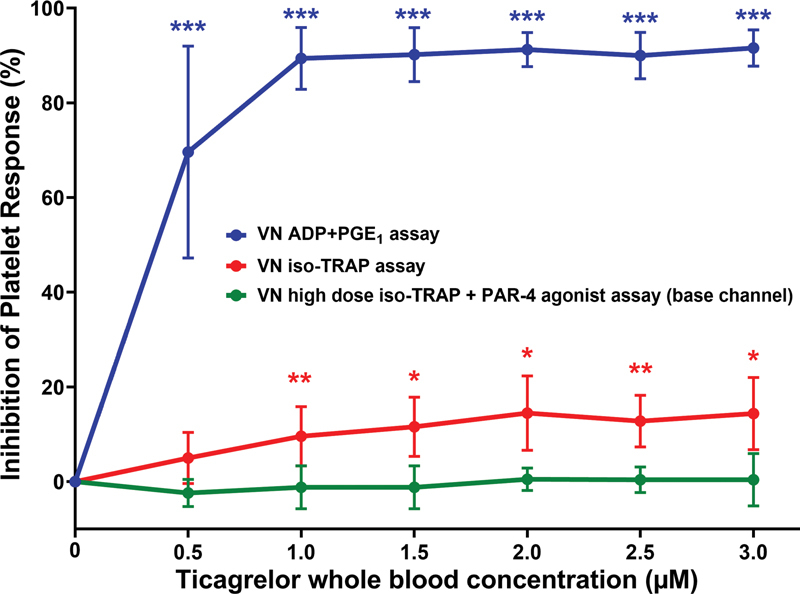
Effect of in vitro addition of ticagrelor on the VN iso-TRAP, ADP + PGE _1_ , and base channel assays, *n* = 5, 34 measurements, data are mean ± SD. All experiments were performed with citrate-anticoagulated blood from healthy donors who were not taking aspirin. * *p* < 0.05 compared with control, ** *p* < 0.01 compared with control, *** *p* < 0.001 compared with control. VN = VerifyNow. PAR-4 AP = PAR-4 activating peptide.

In RUC-4 clinical studies, patients may receive concurrent treatment with aspirin, RUC-4, and a P2Y12 antagonist. In these cases the VN ADP + PGE _1_ assay will be inhibited by both RUC-4 and the P2Y12 antagonist, but not aspirin. Thus, we assessed the potential impact of ticagrelor on the assays. Concentration-response experiments with ticagrelor were performed in VN P2Y12 cartridges. While the ADP + PGE _1_ assay was inhibited even at the lowest concentration, 0.5 µM, the iso-TRAP and base channel assays were minimally affected or unaffected, respectively ( Fig. 5 ). Incubating citrate-anticoagulated whole blood with ticagrelor at 1.5 µM, a concentration representing the sum of the average peak plasma concentrations of ticagrelor and its active metabolite attained in vivo after a 180 mg loading dose (1.96–2.87 µM), 8 9 51 52 resulted in a 90 ± 6% reduction of the ADP + PGE _1_ assay ( *p* < 0.001), 12 ± 6% reduction of the iso-TRAP assay ( *p* < 0.05), and no reduction of the base channel assay ( *p* = 0.53).

#### In the Absence of Ticagrelor, the VN iso-TRAP and Base Channel Assays are Inhibited Similarly by Increasing Concentrations of RUC-4

**Fig. 6:**
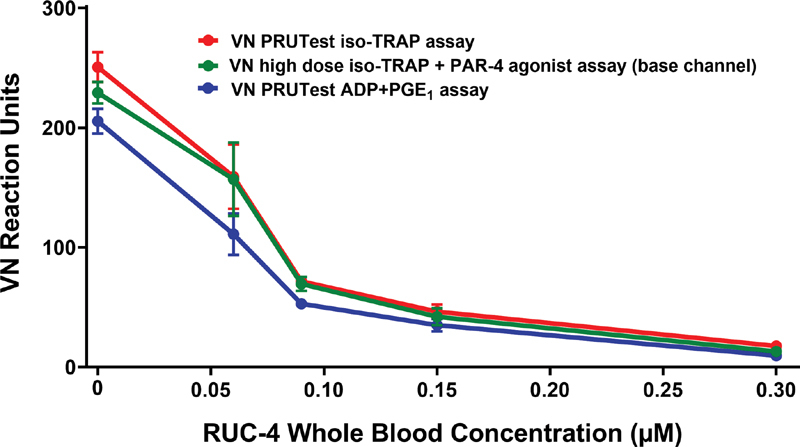
In vitro RUC-4 concentration-response in inhibiting the VN iso-TRAP and base channel assays with blood from healthy volunteers not taking aspirin and anticoagulated with sodium citrate, *n* = 3, 15 measurements, data are mean ± SD. VN = VerifyNow.

We directly compared the sensitivity of the iso-TRAP and base channel assays to inhibition by RUC-4 by adding increasing concentrations of RUC-4 to blood from three donors who were not taking aspirin and measuring the impact on both assays in the same P2Y12 cartridge. The sensitivity of the assays was similar ( Fig. 6 ), with both showing ≥77% inhibition at concentrations ≥0.15 µM. We then determined the base channel reaction unit value that is equivalent to the iso-TRAP 66 reaction unit value that we found corresponds to 80% inhibition of ADP-induced LTA, and found the value to be 62 reaction units by linear regression analysis. Thus, achieving a base channel assay value of 62 reaction units or less indicates that the treatment has achieved the inhibition of platelet function associated with improved in vivo outcomes in PCI for STEMI. 26

## Discussion

Our data indicate that the VN assays may provide valuable PD data during the development of RUC-4 for prehospital therapy of STEMI because: 1. RUC-4 produces *in vitro* and in vivo concentration-dependent inhibition of platelet function measured by the ADP + PGE _1_ , iso-TRAP, and base channel assays. 2. Aspirin does not affect any of the assays, nor does it affect the impact of RUC-4 on the assays. 3. Citrate anticoagulation enhances the sensitivity of the assays to the effects of RUC-4. 4. The ADP + PGE _1_ assay is more sensitive to low concentrations of RUC-4 than the 2 iso-TRAP-based assays. 5. There is a strong correlation between blood concentrations of RUC-4 and αIIbβ3 occupancy, inhibition of ADP-induced LTA, and inhibition of the VN assays. 6. The predicted equivalent of ≥80% inhibition of platelet aggregation induced by 20 µM ADP in PRP made from PPACK-anticoagulated blood, the established standard for predicting αIIbβ3 antithrombotic clinical efficacy, 26 is ≤66 reaction units in the iso-TRAP assay and ≤62 reaction units in the base channel assay. 7. The iso-TRAP assay is much less sensitive to the effects of ticagrelor than the ADP + PGE _1_ assay, but it is partially affected at the highest ticagrelor concentrations achieved in vivo. In sharp contrast, and in accord with studies using the potent P2Y12 antagonist prasugrel, 38 the base channel assay is not affected by even the peak concentrations of ticagrelor likely to be achieved in vivo.

Although our data were developed to facilitate the PD assessment of RUC-4 during its clinical development, our findings may be generalizable to clinical monitoring of RUC-4 therapy if it is approved for treating STEMI. The results may be valuable to the interventionalist performing the PCI, especially if there is consideration of the need for additional αIIbβ3 antagonist therapy on the one hand, or if there is consideration of emergency coronary artery bypass surgery on the other hand. Moreover, the assays can be used in concert to assess when the effect of RUC-4 wears off (using the base channel or iso-TAP assay if a P2Y12 antagonist was not already administered or the base channel assay if a P2Y12 antagonist was already administered), and at that point monitor the onset and/or extent of platelet inhibition by any co-administered P2Y12 antagonist. Since patients will not have baseline VN assay values when treated at first-point-of-contact, we established the optimal iso-TRAP and base channel cut-off value based on absolute reaction units rather than percentage inhibition so that one can assess whether RUC-4 achieved a level of platelet inhibition associated with improved clinical outcomes in patients with STEMI.

The limitations of our study include the following: 1. Our experiments used blood from healthy volunteers or volunteers with stable coronary artery disease, and as such it remains to be established whether the results will be similar in the clinical scenarios of in vivo administration of antiplatelet drugs in patients with acute coronary syndromes. Similarly, results of our experiments with in vitro ticagrelor administration may not be extrapolatable to in vivo use of ticagrelor, or of other P2Y12 antagonists, including prasugrel, which may have benefits over ticagrelor in acute coronary syndromes. 1 Data from the Phase 2A study of RUC-4 administered to patients with STEMI who were also treated with heparin, aspirin, and ticagrelor demonstrated a similar relationship between RUC-4 whole blood levels and inhibition of the VN assays as we observed, supporting the potential applicability of the assays in monitoring STEMI patients, even in the presence of these other drugs. 53 2. Although we did not observe the reported inverse relationship between hematocrit and VN reaction units, the limited range of hematocrits in the participants in our study may have limited our ability to observe a difference. As a result, hematocrit correction may be required in a population with a wider range of hematocrits. 3. We were able to extract the iso-TRAP and base channel data from the commercial VN assays using a computer, but these are not currently reported by the instrument. Thus, to make this information available to clinicians who would like to monitor the antiplatelet impact of RUC-4 or any other αIIbβ3 antagonist alone or in concert with a P2Y12 antagonist, the manufacturer will have to obtain regulatory approval for reporting the results of the iso-TRAP and/or base channel assays. 4. We studied the VN assays because we are familiar with their use, but other point-of-care assays to monitor antiplatelet therapy may also be able to provide similar information. 5. The potential of RUC-4 to produce an increased risk of bleeding is likely to be the major factor in assessing its safety. RUC-4's short duration of antiplatelet effects was designed to reduce the hemorrhagic risks, with its highest grade inhibition of platelet function wearing off before instrumentation to perform PCI and at the time of sheath removal. Since there was no association between bleeding risk and inhibition of an earlier version of the iso-TRAP assay in the GOLD study, 19 VN assays are unlikely to be valuable in assessing hemorrhagic risk.

In summary, the VN assays can be used to assess the pharmacodynamics of RUC-4 during its development and potentially in the future to monitor it or other αIIbβ3 antagonists in the presence of a P2Y12 antagonist. In patients who only receive aspirin and RUC-4 or another αIIbβ3 antagonist, any of the VN assays can provide valuable information. If ticagrelor or another P2Y12 antagonist is administered at the same time as RUC-4 or another αIIbβ3 antagonist, the combination of assays allows for differentiating the relative contributions of each drug.

## Notes

**What is known on this Topic?**

1. Prehospital therapy of ST-elevation myocardial infarction (STEMI) with αIIbβ3 antagonists improves clinical outcomes.
2. RUC-4 is a novel subcutaneous αIIbβ3 antagonist designed for first-point-of-contact therapy of STEMI.
3. The established standard measure of antiplatelet effect is light transmission aggregometry (LTA), but LTA is difficult to perform during STEMI treatment; LTA is also confounded by the presence of multiple antiplatelet agents, such as aspirin, ticagrelor, and an αIIbβ3 antagonist.

**What does this Paper Add?**

1. VerifyNow (VN) ADP + PGE _1_ , iso-TRAP, and base channel (high concentration iso-TRAP + PAR-4 agonist) assays report RUC-4-mediated inhibition in near real-time.
2. RUC-4 dramatically inhibits all 3 VN assays, whereas aspirin does not inhibit the assays, and ticagrelor dramatically inhibits the ADP + PGE _1_ assay, slightly inhibits the iso-TRAP assay, and does not inhibit the base channel assay.
3. VN assays can be used to help differentiate the antiplatelet effects of αIIbβ3 antagonists vs P2Y12 antagonists.
