# Supplementary material for: Application of Auxiliary VerifyNow Point-of-Care Assays to Assess the Pharmacodynamics of RUC-4, a Novel αIIbβ3 Receptor Antagonist
Source: TH Open. 2021 Sep 28;5(3):e449–60. doi: 10.1055/s-0041-1732343 (PMC8478527; doi:10.1055/s-0041-1732343)
Supplement: Supplementary file 1 — Supplementary Material [file 10-1055-s-0041-1732343-s210031.pdf]

Supplementary Material

Supplementary Methods

Preparation of RUC-4 Stock Solutions and Treated Whole Blood for VN Assays

Working stock dilutions of RUC-4 (CeleCor Therapeutics, Del Mar, CA) were made up in sterile 0.9% saline at stock concentrations of the free base between 1.25 and 37.5 µM to achieve final whole blood concentrations between 0.03 and 0.91 µM when 50 µL was added to 2 mL of whole blood. The negative control was prepared by adding 50 µL of sterile 0.9% saline to whole blood. Samples for VN testing that were treated with RUC-4 or vehicle were incubated for 10 minutes at room temperature, as recommended by the manufacturer, before testing.

Preparation of Ticagrelor-treated Whole Blood for VN Assays

A 19 mM ticagrelor stock solution was made by dissolving ticagrelor (Cayman Chemical Company, Ann Arbor, MI) in DMSO and then working stock solutions were prepared in sterile 0.9% saline at concentrations between 40 and 120 µM. To achieve final whole blood concentrations between 0.5 and 3 µM, 50 µL was added to 2 mL whole blood. The negative control was prepared by adding 50 µL of DMSO in sterile 0.9% saline to whole blood (final DMSO concentration 0.01%). Samples were incubated for 30 minutes at 37°C.

Flow Cytometry with PAC1

PPACK-anticoagulated whole blood (3,952 µL) was treated with 28 µL aspirin [made by dissolving acetyl salicylic acid (Sigma Aldrich, Saint Louis, MO) in sterile water and adjusting the pH to 8.1; final concentration 0.3 mM], or control sterile water. Working stock dilutions of RUC-4 were made up in 0.9% saline at concentrations between 2.5 and 125 µM to achieve final whole blood concentrations between 0.01 and 0.63 µM when 20 µL was added to achieve a final volume of 4 mL.

Whole blood was incubated with aspirin and RUC-4, or buffer controls, for 20 minutes at room temperature, after which 2 mL was transferred to a VN tube. A 13.75 µL aliquot was set aside for flow cytometry of whole blood, and the remaining ~2 mL was used to prepare platelet-rich plasma (PRP) for flow cytometry and LTA. PRP was prepared by centrifuging the whole blood at 200 $\times$ g for 6 minutes at room temperature. After removing the PRP, the remaining blood was centrifuged at 1,200 $\times$ g for 8 minutes at room temperature to prepare platelet-poor plasma (PPP). Platelet counts were obtained with the automated counter and samples were adjusted to 3×10^5 platelets/µL with PPP if the PRP count was higher.

Flow cytometry with PAC1 was performed as previously described (reference [24] in manuscript) by adding 13.75 µL of either whole blood or PRP to 6.25 µL of FITC-labeled PAC1 (6.25 µg/mL final concentration; BD Biosciences, San Jose, CA) and either 5 µL of PGE_1 (final concentration 1 µM; Santa Cruz Biotechnology, Dallas, TX) or ADP (final concentration 20 µM; CHRONO-LOG, Havertown, PA) in HEPES-buffered modified Tyrode's solution (HBMT) with 1 mM Mg^{2+}. After incubation for 30 minutes at room temperature, 250 µL of HBMT with 1 mM Mg^{2+} was added to dilute the samples, and flow cytometry was conducted immediately at room temperature (FACSCalibur; BD Biosciences, San Jose, CA). Forward and side light scatter was displayed on logarithmic scales and used to gate the platelet population. Samples were analyzed with the acquisition of 25,000 events. Flow cytometry data were analyzed using BD CellQuest Pro Analysis software. Data are expressed as the percentage PAC1 geometric mean fluorescence intensity observed in the absence of RUC-4.

Statistical Analysis

All continuous variables are reported as mean $\pm$ SD. Continuous variables were plotted on scatter plots and a Pearson correlation coefficient was computed to assess the linear relationship. The multivariate R^2 was computed and a mixed effects logistic regression model was implemented to account for repeated measurements on each participant. Differences in means between 2 dependent or independent groups were analyzed using the student's *t*-test and a 2-tailed p value <0.05 was considered significant. The half maximal inhibitory concentration (IC50) for RUC-4 was determined by fitting a 4-parameter logistic curve to the scatter plot of log [RUC-4 concentration] vs percent inhibition of platelet function. For Receiver Operating Characteristic (ROC) curves, the optimal cut-off points for the VN assays for determining platelet inhibition compared with LTA were identified by calculating the Younden index [J = max(sensitivity + specificity – 1)] by maximizing the distance between the chance diagonal and a point on the ROC curve, which represents the maximum potential of effectiveness for the diagnostic test. ROC analysis included a mixed effects logistic regression model with a random effects for subjects to account for repeated measurements in the same donors (SAS Studio 3.8).

 © 2021. The Author(s).

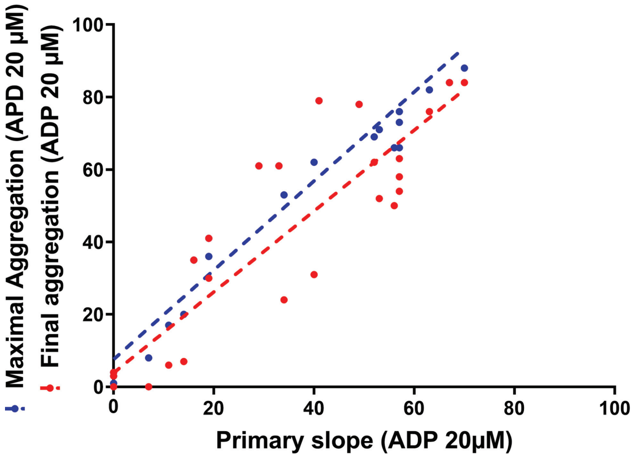

Supplementary Fig. S1 Comparison of the primary slope and the maximal and final platelet aggregation values of all samples tested in the in vitro studies ($n = 6$, 30 measurements). The blue dotted line denotes linear regression of maximal aggregation ($R^2 = 0.92$, $p < 0.001$) and the red dotted line denotes the linear regression for final aggregation at 10 minutes ($R^2 = 0.80$, $p < 0.001$).

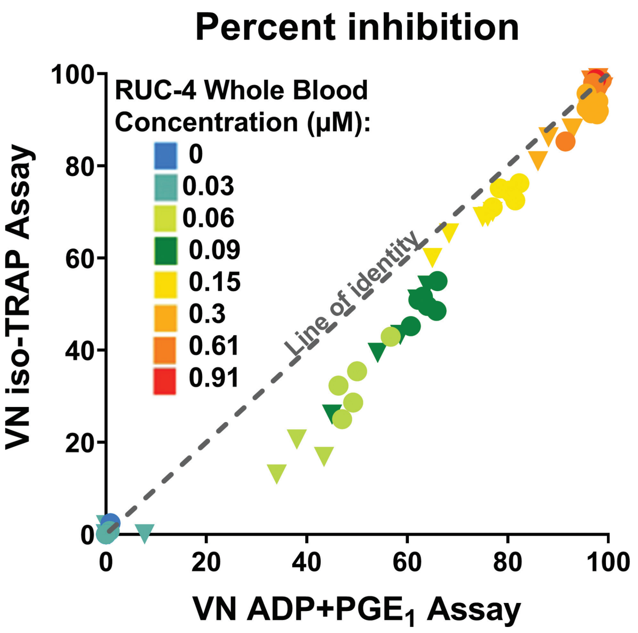

Supplementary Fig. S2 Percent inhibition of response with RUC-4 in citrate-anticoagulated blood tested with the VN PRUTest ADP + PGE_1 assay versus iso-TRAP assay ($R^2 = 0.97$, $p < 0.001$). Circles denote volunteers who were not taking aspirin ($n = 6$; 36 measurements; $R^2 = 0.97$; $p < 0.001$). Triangles denote volunteers who were taking aspirin ($n = 5$; 32 measurements; $R^2 = 0.97$; $p < 0.001$). Dotted line represents the line of identity. VN = VerifyNow.

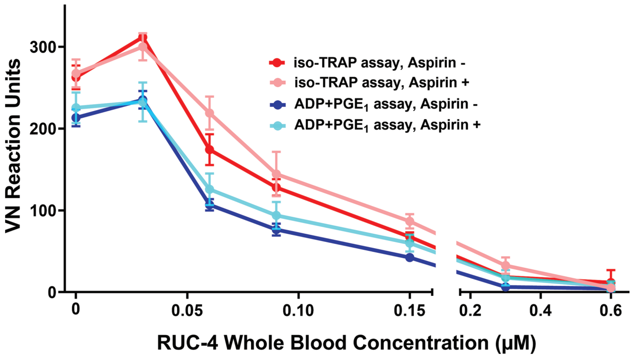

Supplementary Fig. S3 Concentration-response curve of RUC-4 measured by the VN assays in citrate-anticoagulated blood. ($n = 7$ without aspirin and $n = 6$ with aspirin, data are mean ± SD). VN = VerifyNow.

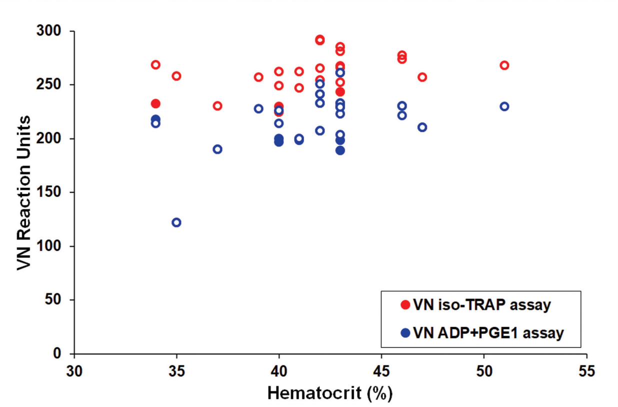

Supplementary Fig. S4 VN assay results as a function of hematocrit in blood tested prior to treatment with RUC-4 or ticagrelor. Filled circles: PPACK-anticoagulated blood ($n = 5$); Open circles: citrate-anticoagulated blood ($n = 21$). iso-TRAP assay: $R^2 = 0.13$, $p = 0.08$; ADP + PGE_1 assay: $R^2 = 0.16$, $p < 0.04$. VN = VerifyNow.

 © 2021. The Author(s).
